# *Solanum lagoense* (Solanaceae, Geminata clade), a new species from Lagoa Santa, Minas Gerais State, Brazil

**DOI:** 10.3897/phytokeys.61.7258

**Published:** 2016-02-25

**Authors:** João Renato Stehmann, Nayara Couto Moreira

**Affiliations:** 1 Instituto de Ciências Biológicas, Departamento de Botânica, Laboratório de Sistemática Vegetal, Universidade Federal de Minas Gerais – UFMG, Av. Antônio Carlos, 6627, Pampulha, Belo Horizonte, CEP 31270-901, MG, Brazil

**Keywords:** Eugene Warming, endemism, assessment of extinction risk, Avaliação do risco de extinção, endemismo, Eugene Warming

## Abstract

A new species of *Solanum* (Solanaceae) from the Geminata clade is described for the Brazilian flora. *Solanum lagoense* Stehmann is only known from Lapinha, a rocky massif located in the Lagoa Santa karst region of Minas Gerais State. The flora of this area, including Solanaceae, was studied in detail in the second half of the 19^th^ century by the Danish botanist Eugene Warming. The species differs from other members of the Geminata clade in Brazil in its geminate leaves of different sizes, simple multicellular trichomes present on the new growth and young stems, short extra-axillary inflorescences with few (1-3) flowers, and its stellate corollas with cucullate and strongly reflexed lobes. Here we present a description, taxonomic comments and a preliminary assessment of conservation status of this critically endangered species.

## Abstract (pt)

Uma nova espécie de *Solanum* (Solanaceae) pertencente ao clado Geminata é descrita para a Flora do Brasil. *Solanum lagoense* Stehmann habita o sub-bosque da Floresta Estacional associada a afloramentos calcários junto à gruta da Lapinha, em Lagoa Santa, Minas Gerais, uma área inventariada em detalhe por Eugene Warming, no século XIX. A espécie é distinta das demais Geminata, por apresentar folhas geminadas de tamanhos desiguais, ramos apicais com tricomas simples, multicelulares, inflorescência extra-axilar, curta, com poucas flores (1-3), corola estrelada, reflexa e cuculada. São apresentadas a descrição da espécie, comentários taxonômicos, bem como a avaliação que sugere o status de Criticamente Ameaçada à espécie.

## Introduction

*Solanum* L. (Solanaceae) is one of the ten largest genera of flowering plants, with 1,250-1,700 species distributed on all continents except Antarctica, but with its highest species diversity in the Neotropics (Frodin 2004; Nee 1999). Among the Solanaceae, *Solanum* is morphologically easy to recognize by its combination of anthers opening by apical pores and a usually evenly 5-lobed calyx. This combination of traits is not shared with any other genera in the family (Hunziker 2001). Important crops are found in the genus, such as potato (*Solanum tuberosum* L.), tomato (*Solanum lycopersicum* L.) and eggplant (*Solanum melongena* L.) that are widely cultivated around the world and contribute to the economies of many countries (Hawkes 1999).

Approximately 272 species of *Solanum* occur in the Brazilian flora, of which 131 are endemic to the country (Stehmann et al. 2015). The highest species richness and endemism are found in the Atlantic rain forest, a biome recognized as a biodiversity hotspot at a global level (Mittermeier et al. 2004). The Atlantic forest originally consisted of an almost continuous strip of land of variable width along the Brazilian coast. Today, this huge forest is represented by remnants and biologically impoverished fragments representing less than 12% of its original cover (Ribeiro et al. 2009). Despite this, several new species in *Solanum* have been described from the Atlantic forest in recent years (Giacomin et al. 2013; Giacomin and Stehmann 2014; Knapp et al. 2015), indicating that the inventory of the Brazilian flora is far from completion (Sobral and Stehmann 2009).

With more than 11,000 species of angiosperms, Minas Gerais State has been recognized as the richest in Brazil (Forzza et al. 2010; Forzza et al. 2012). This floristic richness is associated with the diversity of environments found in Minas Gerais: Atlantic rain forest, Cerrado (savanna-like vegetation), and Caatinga (dry and open thorn scrub). Transition areas between these main vegetation types, mainly those associated with the Espinhaço range, have received special attention since the 19th century, when European naturalists such as Auguste Saint-Hilaire, Carl F. Martius, George Langsdorff, and others travelled in the inner part of the country, describing its flora. Some of these botanists were based in Minas Gerais for long periods of time and explored particular areas in depth; these include the Swede Anders F. Regnell working in Caldas (Concha-Quezada 2011; Dahlgren 1962) and the Dane Eugene Warming in Lagoa Santa (Warming 1908).

Lagoa Santa is a karstic (limestone) region of the Serra de Espinhaço long known for its important paleontological and archeological sites (Berbert-Born 2002). The first scientist to explore its countless caves was the Danish paleontologist Peter Wilhelm Lund (1801-1880), who found many exemplars of the Brazilian megafauna and human fossils. The botanist Johannes Eugenius Bülow Warming (1841-1924) was Lund’s secretary between 1863 and 1866. He collected and took to Europe over 3,000 dried plant specimens. These, in addition to the more than 700 herbarium sheets donated by Lund, are today held in the herbarium of the Natural History Museum in Copenhagen (Gomes 2006). Many of these specimens are nomenclatural types and were cited in the Flora Brasilensis (Martius 1846). Warming distributed herbarium material to many specialists in Europe (Warming 1908). The Solanaceae were worked on by W. P. Hiern who described seven new species and two varieties of *Solanum* (Hiern 1876).

As part of a larger project following Warming’s footsteps, we searched for species with few records and nomenclatural type populations in the same places where Warming collected in Lagoa Santa. During the development of this project, samples of an unusual species of *Solanum* from the Geminata clade were collected. This group is well studied and the Brazilian species of the group have recently been revised (Knapp et al. 2015). We compared our specimens with the others described for the clade and with Warming’s collections kept in the Copenhagen herbarium (C) and other herbaria of the world. We could not match it with any known species and therefore recognize it as new, and describe it here.

## Materials and methods

Specimens of *Solanum* from the following herbaria (acronyms follow http://sciweb.nybg.org/science2/IndexHerbariorum.asp) were examined: BHCB, BM, BR, C, CEPEC, G, HUEFS, K, MBM, PAMG, OUPR, RB, SP, UEC, VIC. We use IUCN (2014) criteria to assess the conservation status of the species.

We collected in Lagoa Santa from December 2014 to March 2015, and focused our efforts on the areas surrounding the rocky massifs such as Lapinha, Sumidouro, and Morro do Baú. These localities present unique environmental conditions due to higher degrees of shade that lead to higher humidity and temperature stability, thus contributing to different species compositions than the surrounding savanna matrix (cerrado).

## Results and discussion

### Solanum lagoense

Taxon classificationPlantaeSolanalesSolanaceae

Stehmann sp. nov.

urn:lsid:ipni.org:names:77153384-1

Figures 1 , 2

#### Diagnosis.

*Solanum lagoense* is similar to *Solanum restingae* S. Knapp, *Solanum amorimii* S. Knapp & Giacomin, and *Solanum psilophyllum* Stehmann & Giacomin but differs from them by its pilose stems and longer fruiting pedicels (> 1.5 cm long).

#### Type.

BRAZIL. Minas Gerais: Município Lagoa Santa, Gruta da Lapinha, Salão dos Bigodes, 19°33'57"S, 43°57'52"W, 716 m, 16 Jan 2015, *N.C. Moreira & R. Gurgel 158* (holotype: BHCB [BHCB021206]; isotype: BM).

#### Description.

Shrub to 1.5 m, rhizomatous, with clonal reproduction; young stems terete, but slightly angled, glabrous or pilose with simple, uniseriate, and recurved trichomes, each with 8–15 cells; new growth always pilose, with stem obviously angled; bark of older stems brown, slightly winged from the leaf bases. Sympodial units difoliate, geminate, the leaves of a pair differing in size, but not usually in shape. Leaves simple; major leaves 5.6–12.4 cm long, 2.2–4.7 cm wide, elliptic, membranous, glabrous on both surfaces, the abaxial surface olivaceous to moss green, the adaxial surface dark green; major veins 7–9 pairs, drying somewhat darker than the lamina and slightly sunken on the adaxial surface, somewhat prominent and lighter on abaxial surface; base attenuate, sometimes slightly asymmetric; margins entire, slightly revolute; apex acute, the tip somewhat blunt; petiole 0.6–1.0 cm long, glabrous; minor leaves 1.6–2.9 cm long, 0.9–1.7 cm wide, differing from major leaves only in size and in having a shorter petiole. Inflorescences 0.2–2 cm long, extra-axillary, arising below the nodes, unbranched, with 1–3 flowers, glabrous; peduncle ca. 3 mm; pedicels 1.4–1.5 cm long, ca. 0.3 mm in diameter, slender, abruptly swollen at the apex, spreading or pendant at anthesis, glabrous, articulated at the base; pedicel scars spaced 0.5–2 mm apart. Buds globose, the corolla strongly exserted from the calyx tube before anthesis. Flowers 5-merous, all perfect. Calyx with the tube ca. 1.0 mm long, broadly conical, the lobes 1.0–1.2 mm long, ca. 1.3 wide, triangular or obtuse, strongly reflexed at anthesis, glabrous adaxially, minutely papillate abaxially, the papillae denser at the tips. Corolla ca. 1.0 cm in diameter, white, stellate, lobed 2/3 of the way to the base, the lobes 1-nerved, ca. 4–5.2 mm long, ca. 2.4–3.6 mm wide, ovate, spreading at anthesis, glabrous, minutely papillate on the margins and the apex, the tips cucullate. Stamens ca. 3.5 mm long; filament tube ca. 0.7 mm long, the free portion of the filaments ca. 0.3 mm long, glabrous; anthers 2.4–2.5 mm long, ellipsoid to slightly obovate, ca. 0.7 mm wide at the base, ca. 0.9 mm wide at the apex, yellow, poricidal at the tips, the pores large and introrse, lengthening to slits with age. Ovary glabrous; style 5–6 mm long, glabrous; stigma not expanded, blunt, the surface minutely papillate. Fruit a subglobose berry, slightly depressed, 1.2–1.3 cm long, 1.3–1.5 cm in diameter, green, darker toward the pedicel, the pericarp not markedly shiny, thick, the mesocarp not juicy; fruiting pedicels 1.7–2.2 cm long, less than 1 mm in diameter at the base, ca. 2 mm in diameter at the apex, gradually expanded to the apex, pendant and hidden under the foliage; fruiting calyx lobes somewhat hyaline, not markedly expanding in fruit, but clearly recurved. Seeds 15–30 per berry, flattened, ellipsoid to irregularly ellipsoid or sometimes ovate-reniform, 3.2–4.0 mm long, 2.8–3.1 mm wide, dark brown, vernicose, with pale incrassate margins, the seed coat obscurely foveolate.

#### Distribution.

Known only from the type locality at the Gruta da Lapinha, Lagoa Santa, Minas Gerais, in southeastern Brazil.

#### Specimens examined

**(paratypes)**. **BRAZIL. Minas Gerais**: Mun. Lagoa Santa, Gruta da Lapinha, Salão dos Bigodes, 19°33'57"S, 43°57'52"W, 716 m, 23 Jan 2015, *J. R. Stehmann & N. C. Moreira 6360* (BHCB). Mun. Lagoa Santa, Gruta da Lapinha, Vale Romano, 10°33'57"S, 43°57'57"W, 912 m, 24 Mar 2015, *J. R. Stehmann et al. 6361* (BHCB). Mun. Lagoa Santa, Gruta da Lapinha, near Vale Romano, 19°33'57"S, 43°57'48"W, 912 m, 24 Mar 2015, *J. R. Stehmann et al. 6374* (BHCB).

#### Ecology.

**Figure 1.**
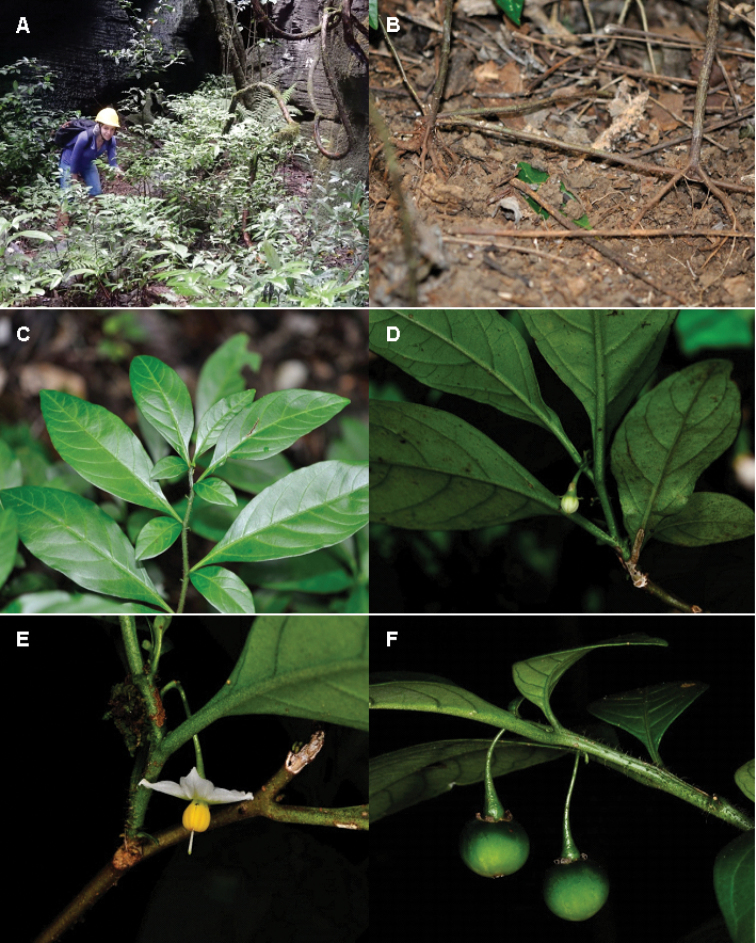
*Solanum lagoense*. **A** Habit and Nayara Moreira standing close to plants **B** Clonal reproduction via rhizomes **C** Geminate leaves of different sizes **D** Bud **E** Flower showing the cucullate and spreading corolla lobes **F** Fruits showing the markedly recurved calyx lobes. **A, C, D** *Stehmann et al. 6360*; **B, E** *Stehmann et al. 6361*; **F** *Stehmann et al. 6374*.

*Solanum lagoense* grows on well-drained soils in the understory of the seasonal forest (Floresta Estacional Semidecidual) that covers the entrance of caves as well as the canyons and blind valleys associated with the carbonatic rocky massifs of Lagoa Santa. This specific habitat is very stable with respect to temperature and humidity throughout the year, in comparison with Cerrado, the typical vegetation matrix in the region. An extensive subterranean system of rhizomes connects individuals in the populations we have sampled (Figure 1B), indicating that this species is capable of vegetative reproduction. We observed no bees visiting the flowers. The green fruits, hanging and hidden under the foliage, suggest dispersal by bats that inhabit the caves, but the natural history of this species is in need of detailed study.

**Figure 2.**
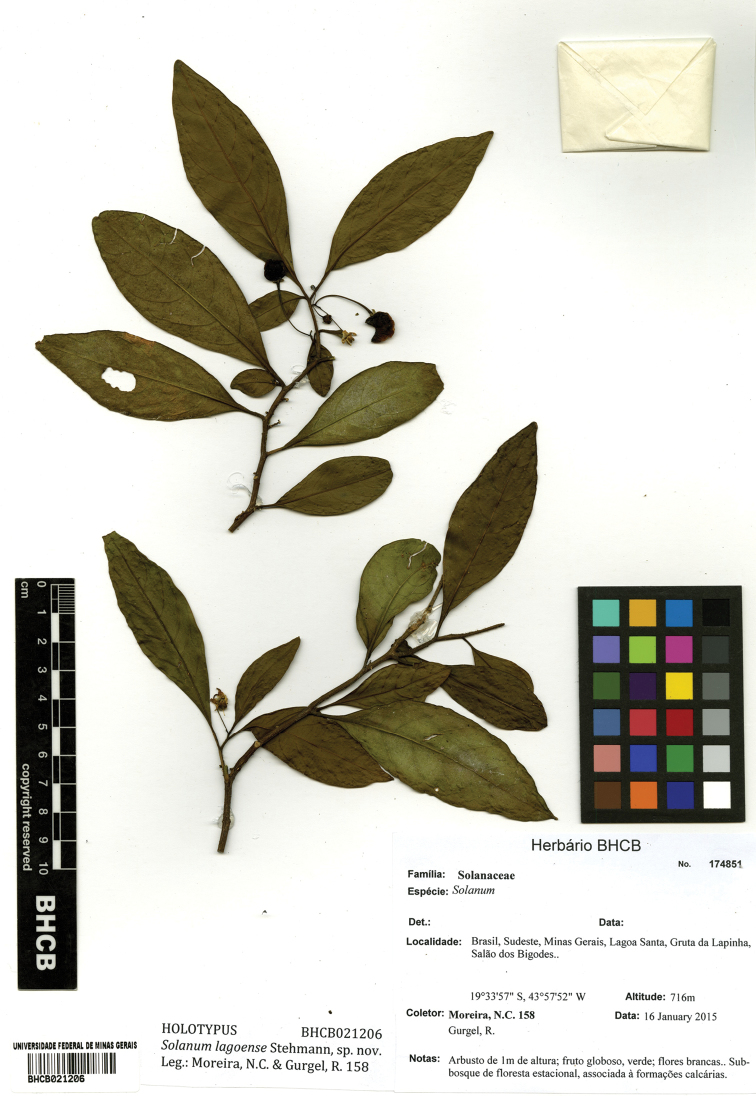
Holotype specimen of *Solanum lagoense* (*N.C. Moreira & R. Gurgel 158* [BHCB021206]).

#### Phenology.

Flowering specimens were collected in January, occasionally in March, while fruiting material was seen in January, February, and March.

#### Etymology.

The name refers to Lagoa Santa, a Brazilian locality where two important Danish researchers, Peter Lund and Eugene Warming, worked in the mid 19th century. Warming started his botanical career here studying the Cerrado flora and its ecological relationships. Nowadays he is recognized as one of the Fathers of Ecology.

#### Preliminary conservation status

**(IUCN 2014).**
Critically Endangered (CR) B1, 2 a, b(ii, iii, iv). This species is known from a single locality, the Gruta da Lapinha, included in the Parque Estadual do Sumidouro, a protected area that encompasses 52 caves. There is an increasing human pressure in its microhabitat near the base of the limestone walls, where many climbing routes are in constant use. The limestone outcrops have being mined for decades, drastically reducing the habitat of this species. The surrounding landscape is changing very quickly with the growth of the municipality of Lagoa Santa, influenced by the Vector North project that fostered the expansion of the metropolitan region of Belo Horizonte, the capital of the state (Auler and Piló 2015). All of these threats support an assessment of Critically Endangered. Efforts to locate new populations in the conservation unit, as well as in similar habitats associated with caves outside it are needed.

#### Notes.

*Solanum lagoense* is a small shrub with entirely glabrous leaves, short inflorescences, few small flowers, and green fruits that are hidden below the foliage. These characters are common in species belonging to Geminata clade, a group that is highly diverse in the Atlantic forest (Knapp 2002; Knapp et al. 2015). *Solanum lagoense* is similar to *Solanum restingae*, *Solanum amorimii* and *Solanum psilophyllum* Stehmann & Giacomin due to its glabrous and geminate, but not dimorphic leaves. The latter three species are distinguished by their glabrous stems, even on the new growth, and short fruiting pedicels (up to 1.5 cm long), whereas *Solanum lagoense* has clearly pilose young stems and longer fruiting pedicels. *Solanum restingae* is endemic to Bahia and Espírito Santo states and has a strongly winged stem and basally attenuate leaves. *Solanum amorimii* grows in southern Bahia, but also in far northeastern Minas Gerais; its stem is not winged, the leaves are somewhat auriculate at the base. Despite its occurrence in the region of the Serra do Cipó and the Iron Quadrangle relatively close to Lagoa Santa, *Solanum psilophyllum* has longer petioles (>1.5 cm) and leaves (>10 cm) and more flowers per inflorescence (5–8).

Other species belonging to the Geminata clade recorded in the southern part of Espinhaço mountains in Minas Gerais are *Solanum verticillatum* Knapp & Stehmann, *Solanum gnaphalocarpon* Vell., *Solanum intermedium* Sendtn., and *Solanum warmingii* Hiern, the last three collected by Warming in Lagoa Santa and cited or described by Hiern (1877). It is noteworthy that after Warming’s intensive collecting efforts in Lagoa Santa (1863-1866), including the Lapinha and Sumidouro limestone outcrops, few new species have been described in the last decades. This can be due to Warming’s extensive collecting or to the fact that few researchers have been collected recently in the area. Most collecting efforts in the southern Espinhaço chain have been concentrated in rocky quartzite fields (campos rupestres) found in high altitude areas (above 900 m) that houses one of the richest floras of the Neotropical region, almost half of the species endemic (Echternacht et al. 2011; Giulietti and Pirani 1997).

At first glance, *Solanum lagoense* also resembles species belonging to *Solanum inornatum* clade, but the trichomes, leaf arrangement, and number of seeds are quite distinct. While *Solanum inornatum* group shows trichomes with few cells (up to 4), geminate leaves differing in form, and translucent fruits with few seeds (up to 10) (Giacomin 2015), *Solanum lagoense* has multicellular soft trichomes, with more than eight cells, geminate leaves equal in form and hard, green berries with more than 15 seeds per fruit.

The clonal reproduction in *Solanum lagoense* is noteworthy. All individuals of the population studied have horizontal rhizomes below the leaf litter, linking all the plants together, similar to other members of the Geminata clade such as *Solanum arboreum* of northern South America (Knapp 2002) and *Solanum psilophyllum* of the southern Espinhaço range in Minas Gerais. In Solanaceae, vegetative reproduction is common in the tuber-bearing potatoes (Hawkes 1990; Spooner et al. 2014), and has also been reported in weedy species of the Leptostemonum clade growing in open places or forest margins such as *Solanum viarum* Dunal, *Solanum palinacanthum* Dunal, *Solanum guaraniticum* A. St.-Hil., and *Solanum paniculatum* L., all common species of southeastern Brazil (Mentz and Oliveira 2004). It appears clonal reproduction is widespread across *Solanum*, and it has been reported in the Cyphomandra, Morelloid, Dulcamaroid, and Brevantherum clades (Giacomin and Stehmann 2014; Vallejo-Marín and O’Brien 2007). The extent of this habit and reproductive mode is not well-documented in Neotropical solanums largely because the underground parts are rarely collected or even observed in these woody plants (see Knapp 2002).

Lagoa Santa is considered an example of a well-catalogued site. Warming compiled a thorough collection listing 2,593 plant species (Warming 1908). Our discovery of this new species in Lagoa Santa strengthens the claim for more floristic and taxonomic inventories in Brazil, not only in poorly collected areas such as Amazonia (Sousa-Baena et al. 2014), but also in “well-studied” areas. In-depth floristic inventories in places with difficult access or with distinct and poorly documented microclimatic conditions, have often resulted in discoveries of new rare and endemic species, even in what appear to be well-catalogued sites.

## Supplementary Material

XML Treatment for Solanum lagoense
